# Effect of transdermal magnesium cream on serum and urinary magnesium levels in humans: A pilot study

**DOI:** 10.1371/journal.pone.0174817

**Published:** 2017-04-12

**Authors:** Lindsy Kass, Andrea Rosanoff, Amy Tanner, Keith Sullivan, William McAuley, Michael Plesset

**Affiliations:** 1 University of Hertfordshire, Hatfield, Hertfordshire, United Kingdom; 2 Center for Magnesium Education & Research, Pahoa, Hawaii, United States of America; Indiana University Richard M Fairbanks School of Public Health, UNITED STATES

## Abstract

**Background:**

Oral magnesium supplementation is commonly used to support a low magnesium diet. This investigation set out to determine whether magnesium in a cream could be absorbed transdermally in humans to improve magnesium status.

**Methods and findings:**

In this single blind, parallel designed pilot study, n = 25 participants (aged 34.3+/-14.8y, height 171.5+/-11cm, weight 75.9 +/-14 Kg) were randomly assigned to either a 56mg/day magnesium cream or placebo cream group for two weeks. Magnesium serum and 24hour urinary excretion were measured at baseline and at 14 days intervention. Food diaries were recorded for 8 days during this period. Mg test and placebo groups’ serum and urinary Mg did not differ at baseline. After the Mg^2+^ cream intervention there was a clinically relevant increase in serum magnesium (0.82 to 0.89 mmol/l,p = 0.29) that was not seen in the placebo group (0.77 to 0.79 mmol/L), but was only statistically significant (p = 0.02)) in a subgroup of non-athletes. Magnesium urinary excretion increased from baseline slightly in the Mg^2+^ group but with no statistical significance (p = 0.48). The Mg^2+^ group showed an 8.54% increase in serum Mg^2+^ and a 9.1% increase in urinary Mg^2+^ while these figures for the placebo group were smaller, i.e. +2.6% for serum Mg^2+^ and -32% for urinary Mg^2+.^ In the placebo group, both serum and urine concentrations showed no statistically significant change after the application of the placebo cream.

**Conclusion:**

No previous studies have looked at transdermal absorbency of Mg^2+^ in human subjects. In this pilot study, transdermal delivery of 56 mg Mg/day (a low dose compared with commercial transdermal Mg^2+^ products available) showed a larger percentage rise in both serum and urinary markers from pre to post intervention compared with subjects using the placebo cream, but statistical significance was achieved only for serum Mg^2+^ in a subgroup of non-athletes. Future studies should look at higher dosage of magnesium cream for longer durations.

**Trial registration:**

ISRCTN registry ID No. ISRTN15136969

## Introduction

Mineral elements, such as magnesium (Mg^2+^), are required by the human body in modest amounts for the maintenance of health and the development of optimal functioning[1]). Mg^2+^ is an important mineral element and it is the fourth most abundant cation in living organisms, with Mg^2+^ being a cofactor to over 325 enzymatic reactions within the body [2]. Around 99% of total body Mg^2+^ is located in the bone, muscles and non-muscular soft tissue [3]. Mg^2+^ supplementation has been shown to significantly improve blood pressure [4–6] as well as modifying vascular tone by regulating endothelium and smooth muscle cell function [4].

To maintain an adequate Mg^2+^ status humans must consume Mg^2+^ at regular intervals [7]. The daily recommendation for Mg^2+^ is controversial, as the literature is conflicting and varies between countries, although values of ≥ 300 mg/day are usually reported for healthy adults with adjustment for age, sex and nutritional status [7].

Oral magnesium supplementation has been shown to affect various parameters, such as blood pressure [5,8], immune function [9], cardiovascular system [10] and metabolic syndrome [11]. A recent meta-analysis by Zhang et al (2016) in the Journal of Nutrition [12] found that in a healthy population group there was a dose dependent increase in both serum Mg concentration and urinary Mg excretion with supplemental oral magnesium intake ranging from 250–500mg/day. Kass, Weekes and Carpenter (2012) identified that a supplement of >370 mg/day of Mg^+^ shows greater efficacy than a lower dose in improving blood pressure and that magnesium supplementation gives a dose dependent response with regards to blood pressure [5].

An alternative method of perhaps attaining recommended magnesium intakes might be through topical application. Current formulations include magnesium oils and trans-dermal creams, from which the magnesium may be absorbed across the skin and into the systemic circulation. However, in contrast to gastrointestinal epithelium, a primary function of the skin is to act as a barrier, which restricts the absorption of exogenous chemicals into the body. The barrier function of the skin is thought to lie predominately in the outermost layer of the epidermis, the stratum corneum. The stratum corneum is thought to largely present a hydrophobic barrier to the absorption of transdermal creams. The dermis, immediately below the epidermis, contains the blood vessels that are able to transport substances that have permeated the skin into the systemic circulation.

Although less studied than organic molecules, metal ions are known to be able to cross the skin, with the literature having focussed on metals that are known to cause irritant/toxic effects [13]. The lower resistance to permeation of the skin appendages, skin structures that serve a particular function including sensation, lubrication and heat loss, and the ionised nature of metals means that their permeation across these skin appendages is considered to be the most likely route of absorption [13,14]. However, the low surface area available for this in human skin means that metal ion absorption across skin is expected to be relatively low. Therefore, it has been questioned that a transdermal route of administration might provide sufficient Mg^2+^ absorption to help meet systemic Mg^2+^ requirements. This investigation aims to determine if transdermal absorption of Mg^2+^ from a topical cream occurs “in vivo” in humans.

To date, no study has investigated the absorbency of transdermal magnesium cream in human subjects. Commercially available Mg^2+^ topical applications range from 75mg to 400mg depending on the dosage recommended by manufacturers. This ranges from 5–30 sprays of magnesium oil or 2–4 teaspoons of magnesium cream, which can be applied in one application or throughout the day. Disappointingly, many commercial topical creams and oils do not state the concentration of magnesium in the product.

This study was designed as a first time, pilot study to ascertain whether such a topical Mg^2+^ preparation might affect urinary or serum Mg^2+^. Since less than 1% of magnesium is contained in the blood, assessment by serum status may be problematic [3]. It is often considered that 24-hr urine excretion of Mg^2+^ may be a better indicator of intestinal absorption than serum concentration; however, urinary Mg^2+^ excretion is also highly variable and it is questionable whether it can be used reliably to assess an individual’s Mg^2+^ status [15]. However, serum Mg^2+^ can reflect a longer term dietary Mg^2+^ status over weeks or months whilst urinary Mg^2+^ can be a better marker of one’s recent dietary intake [16]. These studies using serum and urinary Mg^2+^ markers in dietary Mg^2+^ research could not be assumed to be helpful in the design of this study. Djurhuus et al,[17], however, reported that although it is unlikely that a single determination of serum Mg^2+^ can be used in assessing whole-body Mg^2+^ status in an individual, serial determinations of serum Mg^2+^ might prove useful as an indicator of changes in whole body Mg^2+^ status. These authors also found that 24-hr urinary Mg^2+^ excretion is unlikely to be a reliable measure of whole body Mg^2+^ status and is not a good marker to measure changes in whole body Mg^2+^ status. Nonetheless, we decided to use 24-hr urinary Mg^2+^ as well as serum Mg^2+^ in this pilot study.

Therefore, the purpose of this pilot study was to investigate whether a 56 mg/day dose of magnesium in a cream, applied transdermally to humans, would affect either serum magnesium levels or urinary excretion over a two-week period and to measure effect, if any, and variance to inform a properly powered future study, if warranted.

## Methods

### Recruitment

Subject recruitment started April 2014 and data collection and follow-up was completed by February 2015. The study was not registered on a CT Database at the time of subject recruitment as trial registration in these kinds of studies is not commonly practiced. The trial was subsequently registered in order to comply with publication requirements according to the WHO guidelines. The authors confirm that all ongoing and related trials for this intervention are registered.

### Participants

Twenty-five healthy adults (female = 13 male = 12) aged 34.3+/-14.8y, height 171.5+/-11cm, weight 75.9 +/-14 Kg, were recruited from the staff and student population of the University of Hertfordshire and word of mouth to local residents and randomly assigned into either a magnesium cream or placebo cream group by random allocation. Randomisation was determined by allocation to a group selected from a box with equal amounts of paper stating either placebo or magnesium and selected at time of recruitment. The trial was single blind with only the lead investigator being aware of the content of the cream. One participant dropped out of study before completion. Participants were excluded if they were taking magnesium supplementation in any form within a month of recruitment onto the trial, were under the age of 18y or above the age of 60y.There were no height or weight restrictions, Written informed consent was completed and ethical approval was granted by the University of Hertfordshire Health and Human Science Ethics Committee on 14^th^ April 2014. Blood collection and blood and urine analysis was undertaken at the University of Hertfordshire Human Physiology Laboratory.

Dietary magnesium intake was below the RNI in one of the placebo participants and three of the magnesium participants. This was not considered a bar to inclusion into the study as any change in serum or urinary Mg2^+^ from the cream could still be shown. High levels of physical exercise have been shown to deplete human Mg^2+^ status ([18]. Four participants were considered “athletes” as they engaged in at least 2hrs of physical exercise at least 5 days per week during the study, three who were assigned to the Mg^2+^ intervention group and one assigned to receive placebo. All other 20 participants who completed the study were considered “non-athletes”, i.e. engaging in less than 2 hrs physical exercise per day for no more than 3 days per week during the study. Participants were instructed not to exercise 24h before blood draws, but “athletes” may have disregarded this instruction, so results were statistically analysed in two ways: 1. “all subjects”, including both athletes and non-athletes (n = 24 who completed the study) and 2. “non-athletes” (n = 20) which excluded the 4 athletes. Baseline serum and urine Mg^2+^ concentrations as well as dietary Mg^2+^ recorded in the randomised intervention and placebo groups did not differ significantly from each other (P<0.05) (Tables 1 & 2) in either the “all subjects” or “non-athletes” groupings.

**Table 1:** Demographic and baseline serum, urine and dietary magnesium (mean +/- s.d. [range]) for all subjects, athletes plus non-athletes.

	Magnesium group N = 14	Placebo group N = 10
Age (years)	34.8±15.3	36.6±14.6
Height (cm)	171.2±8.9	173.8±12.4
Weight Kg)	77.5±15.9	75.6±11.5
Serum (mmo/l)	0.82±0.18 [0.62–1.24]	0.773±0.16 [0.56–1.16]
Urine (mmol/24h)	4.07±1.62 [1.30–7.00]	4.6±2.1 [2.7–9.0]
Dietary 9Mg)	294.9±93.8 [176–496]	329.1±91.9 [196–482]

**Table 2:** Demographic and baseline serum, urine and dietary magnesium (mean +/- s.d. [range]) for non-athletes only.

	Magnesium group N = 11	Placebo group N = 9
Age (years)	34.6±13.7	38.5±14.6
Height (cm)	169.7±9.4	175.0±12.5
Weight (Kg)	75.6±17.1	75.8±12.1
Serum (mmol/l)	0.75±0.13 [0.62–1.07]	0.73±0.09 [0.56–0.85]
Urine (mnol/24h)	4.08±1.34 [2.02–6.11]	4.80±2.1 [2.9–9.0]
Dietary (mg)	285.8±82.2 [177–469]	331.0±97.3 [196–482]

### Baseline measurements

Baseline data consisted of a 24 hour urinary collection to assess baseline magnesium excretion and venous bloods to assess serum magnesium levels. (Tables 1 and 2). Urine was collected from the second urinary passing of the day until and including the first urinary passing of the next day. Blood collection could take place at any time of the day to suit the participants but each participant had to return at that same timeslot for the second blood collection. No food was allowed for the 3 hours before blood collection although water was allowed.

### Dietary analysis

Each participant recorded a 4-day food diary (3 midweek days and 1 weekend day) prior to the intervention plus a second 4-day food diary at the end of the 12–14 day period, giving a total of 8 days dietary analysis over the period of the intervention for each participant. This was analysed for Mg^2+^ intake using Diet Plan 6 software (Forestfield Software Ltd., West Sussex, UK). (Tables 1 and 2).

### Intervention

After baseline measurements were taken, participants were randomly assigned to either the Mg^2+^ Cream or a placebo control cream and were instructed to apply 2 x 5ml spoonfuls of cream per day for two weeks. The resulting daily Mg^2+^ dose received by subjects in the Mg^2+^ group consisted of 56mg of Mg^2+^. This was manufactured, in the course of research and development, for the Center for Magnesium Education & Research by Urist Cosmetics of Vancouver, B.C. Canada. For full ingredients see S1 Text Ingredients list.

The placebo was a commercially available aqueous cream containing no magnesium (by analysis) and creams were packaged identically. Instructions to participants suggested that Mg^2+^ or placebo cream be applied to the stomach and torso in the first instance and then spread down to the legs. Time of day was not important, but no showering or washing could take place for a minimum of 3 hours after application. After 12–14 days, final urine and blood samples were collected. The cream was applied up to and including the day of the final urine and blood collection. Participants were instructed to stop use of the cream if there was any adverse reaction, of which there were none. At the end of the trial compliance was ensured from verbal communication as well of collection of the trans-dermal cream container to ensure full usage.

### Sample collection

Serum blood samples were collected by venepuncture from the median cubital, basillic or cephalic vein. Serum separator vacutainers were inverted 10 times before being left to rest for 30 minutes. Subsequently, samples were centrifuged at 3000 rpm for 10 minutes. Serum was checked to be free from haemolysis and was immediately pipetted and frozen at -80°C for subsequent analysis

Urine was collected into 3 litre collection vessels over 24 hours. Urine was then decanted into a measuring vessel and volume of urine was recorded. The measuring vessel was then placed on a magnetic stirrer at 100rpm. To re-suspend the Mg^2+^, the pH was lowered to 3–3.25 by adding 5 M hydrochloric acid. Duplicate 1mL aliquots were frozen at -80°C for batch analysis.

### Analyses

Urine and serum samples were analysed by colorimetric assay for magnesium (RX MONZA, Randox Laboratories Limited, County Antrim, United Kingdom). The machine was calibrated according to the manufacturer's instructions and results were calculated from the standard concentration curve generated using the manufacturer’s calibration standard. Low urinary and serum Mg^2+^ were determined to be below 3.0mmol/24h and 0.65mmol/l respectively [19].

All urine and serum samples were frozen and stored at -80°C for between 4–12 weeks.

### Data analysis

Serum and urinary Mg^2+^ data for both pre and post intervention were analysed for skewness and normality prior to statistical analysis. All data passed the normality test so a standard two tailed, paired t-test was used to compare baseline to post-intervention values (serum and urinary Mg) for within group analysis.

Statistical analysis was conducted using SPSS V22 (IBM, New York, USA), with P value value of ≤0.05 accepted as statistically significant.

## Results

**Fig 1:**
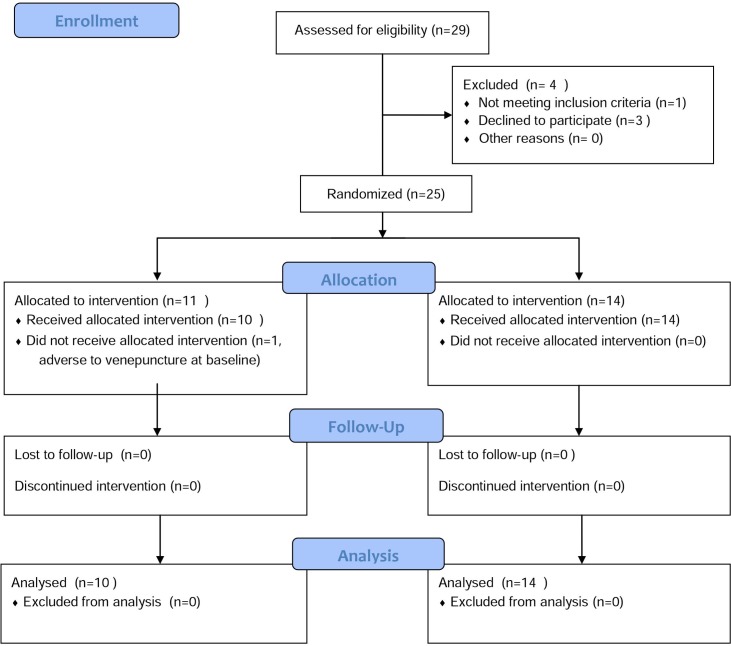
CONSORT flow diagram, showing inclusion and exclusion of participants in the study.

Of the 29 subjects recruited, 1 was excluded for not meeting the criteria and 3 declined to participate after baseline data was collected. Fourteen participants were allocated to the magnesium intervention group and completed the trial. Eleven were allocated to the placebo intervention from which one dropped out due to adverse reaction to the venepuncture at the baseline blood collection before cream had been administered. Fig 1 depicts the inclusion and exclusion of the participants in the study.

### Urinary and serum response to trans-dermal magnesium application

Mean serum and 24-hour urinary Mg^2+^ concentrations were obtained for all participants before and after a 2-week application period of the magnesium or placebo trans-dermal cream. Baseline (Tables 1 & 2) serum Mg^2+^ values were below normal reference values (0.65mmol/l) in one magnesium intervention participant and one placebo participant (both non-athletes), whilst the baseline 24-hr urinary Mg^2+^ excretion was below normal (3.0mmol/24h) in two placebo participants (one non-athlete, one athlete) and three magnesium participants (two non-athletes, one athlete); these were different participants for both the serum and urine. Of the five participants with low urinary excretion, three had below the RNI for dietary Mg^2+^ intake however this was not the case for the low serum Mg^2+^ subjects.

### All participants, Table 3

There was no statistically significant effect of Mg^2+^ cream on either serum or urinary Mg^2+^ status. However, after the Mg^2+^ cream intervention there was a clinically relevant increase [11, 19] in serum magnesium (0.82 to 0.89 mmol/l) that was not seen in the placebo group (0.77 to 0.79 mmol/L), but this was not statistically significant (p = 0.29). Similarly, there was a slight increase in magnesium urinary excretion in the Mg^2+^ group but again no statistical significance (p = 0.48). A percentage increase of 8.54% for serum Mg^2+^ and 9.1% in urinary Mg^2+^ was seen in the Mg^2+^ group while for the placebo group these figures were smaller, i.e. +2.6% for serum Mg^2+^ and -32% for urinary Mg^2+^. In the placebo group both serum and urine concentrations showed no statistically significant change after the application of the placebo cream.

**Table 3:** Influence of Trans-dermal Magnesium vs placebo cream application on Serum and Urinary Magnesium Concentrations (mean +/- S.D.) for all subjects, athletes plus non-athletes.

	Serum Concentrations,	(mmol/L)	Urinary Excretion	(mmol/24h)
Intervention	Magnesium (n = 14)	Placebo (n = 10)	Magnesium (n = 14)	Placebo (n = 10)
Pre	0.82±0.18	0.77±0.16	4.07±1.62	4.6±2.1
Post	0.89±0.18	0.79±0.18	4.44±1.56	3.12±1.27
%Change	+8.54%	+2.6%	+9.1%	-32%

### Non-athletes, Table 4

For non-athlete participants (n = 11 in Mg group plus n = 9 in placebo group), there was a statistically significant rise (P = 0.02) in serum Mg^2+^ in the intervention group which was not seen in the placebo group. Serum Mg^2+^ in the intervention showed a % change of +22.7% while that in the placebo group showed a +4.11% rise. Urinary Mg^2+^ did not show any significant change in either Mg^2+^ or placebo group although a large non-significant negative change could be seen in the placebo group (-32.50%) when compared with the 11.3% increase in the intervention group.

**Table 4:** Influence of Trans-dermal Magnesium vs placebo cream application on Serum and Urinary Magnesium Concentrations (mean +/- S.D.) for non-athlete subjects.

	Serum Concentrations,	(mmol/L)	Urinary Excretion	(mmol/24h)
Intervention	Magnesium (n = 11)	Placebo (n = 9)	Magnesium (n = 11)	Placebo (n = 9)
Pre	0.75±0.13	0.73±0.09	4.08±1.34	4.80±2.10
Post	0.92±0.18*	0.76±0.17	4.54±1.75	3.24±1.28
%Change	+22.7%	+4.11%	+11.27%	-32.50%

* P<0.02 compared with “pre-serum Mg^2+^ value

## Discussion

### Transdermal Mg^2+^ effect on serum Mg^2+^

Previous study of humans have shown that serial determinations of serum Mg^2+^ can prove useful as an indicator of changes in whole body Mg^2+^ status[17]. From this study, 56 mg/day Mg^2+^ applied as transdermal cream for 12–14 days had no statistically significant effect on serum concentration in this small (n = 25) human study when both athletes and non-athletes were included in the statistical analyses. However, a trend towards a rise in serum Mg^2+^ in the Mg^2+^ group could be seen with an increase of 0.07mmol/l, a clinically relevant rise in a measurement that is greater than many previous studies and a rise that would take months to show change with oral Mg^2+^ therapy [11, 19]. Zhang et al., [11] reported that in 41 trials, 941 participants receiving a mean oral Mg supplement of 365 mg/day for a median of 12 weeks showed a mean rise of 0.05 mmol/L circulating Mg (0.78 to 0.83 mmol/L). A meta-analysis of 27 trials by Zhang et al [8] investigating oral Mg^2+^ supplementation, showed a significant (p<0.001) rise in serum Mg with 200 mg/day oral Mg supplement or one month supplement duration but that higher doses (≥ 300 mg/day) or durations of supplementation (≥2 months) were required to achieve a mean rise of 0.05 mmol/l in serum Mg^2+^. Additionally, studies included in the Zhang et al [8] meta-analysis show a baseline mean C.V. for the 27 serum Mg^2+^ studies of 9.3% for Mg groups and 10.8% for placebo groups, i.e. as little as half of the variance for baseline serum Mg^2+^ measurements in this study (C.V. = 21.9% and 17.3% for Mg^2+^ groups, all subjects and non-athletes respectively; C.V. = 20.7% and 12.3% for placebo groups, all subjects and non-athletes respectively). This pilot study of all subjects, both athletes and non-athletes, shows that transdermal Mg^2+^ may possibly influence serum Mg^2+^ in a relatively short time frame (12–14 days), but a higher concentration of Mg^2+^ cream, a larger number of subjects given the serum Mg^2+^ variance, and perhaps a longer study is required to make any real conclusion.

Although participants were told to refrain from exercise for 24 hours before blood and urinary collection, 4 participants were undertaking regular high intensity training, the effects of which may affect Mg^2+^ parameters that can last longer than 24 hours [18]. When the data from these four subjects were removed from the statistical analysis a significant effect of the Mg^2+^ cream on serum Mg^2+^ concentrations could be seen.(p = 0.02). Additionally, the %rise in serum Mg^2+^ in the non-athletes (+22.7%) was much larger than that shown in all subjects, i.e. both athletes and non-athletes (+8.54%). This is too small a sample size to reach a firm conclusion but this increase in percentage change in the non-athlete intervention group may be due to an additional uptake of Mg^2+.^from the cream which may have been utilised during exercise or for replenishment of Mg^2+^ stores rather than being transferred to serum in the athletic participants. This is an area of interest for further study.

Further, this analysis of the serum Mg data for the non-athletes in the intervention group showed a mean increase from 0.75 to 0.92 mmol/l which may have clinical relevance in particular with relation to cardiovascular disease. In a meta-analysis by Del Gobbo et al (2013)[20], it was found that a rise of 0.2 mmol/L circulating Mg was associated with a 30% lower risk of cardiovascular disease and fatal ischemic heart disease. In addition, Lutsey et al.(2014) [21] found that after 20+ yrs follow up serum Mg showed a linear inverse association with the risk of incident heart failure. Relative to those in the highest category of serum Mg, those in the lowest category were at 2.58 times greater risk of incident heart failure after demographic adjustments. In these quintiles, the lowest serum Mg quintile was 0.7 mmol/L, the second was 0.75, the third was 0.8, the 4th was 0.85 and the highest was 0.9 mmol/L, i.e. each quintile was 0.05 mmol/l higher than the next lowest quintile (these results were converted from mEq/L by dividing by 2 to attain mmol/L). Our results show a mean rise of 0.05 mmol/L serum Mg^2+^ with daily application of transdermal Mg^2+^ for only two weeks, and when considering this Lutsey[21] study and the Del Gobbo[20] results, our finding suggests a possibly significant favourable impact of transdermal Mg on risk of heart failure that needs full study.

### Transdermal Mg effect on urinary Mg^2+^

Previous study of humans has suggested that 24-hr urinary Mg^2+^ excretion cannot be used as a measure of changes in whole body Mg^2+^ status [17].

Upon analysis for all subjects, as well as for only non-athletes, use of the transdermal Mg^2+^ cream showed no significant rise in urinary Mg^2+^, a measurement that reflects short term intestinal absorption of Mg^2+^. However, subjects in the Mg^2+^ group showed slight rises in urinary Mg^2+^ (+9 to 11%) while those in placebo group showed a substantial decrease in urinary Mg^2+^ (-32%). Possibly the decreased urinary Mg^2+^ excretion in the placebo cream group represents more active physiological Mg^2+^ retention processes that are not apparent in the Mg cream group ([15,16]. It has been suggested that 24-hour urine excretion of Mg^2+^ may be a better indicator of tissue status than the serum Mg^2+^ concentration, but it is highly variable and it is questionable whether it can be used to reliably assess a given individual’s Mg^2+^ status.

The trans-dermal cream contained 56 mg of Mg^2+^ administered daily. This is at the lower end of creams sold commercially. The recommended dose of the few commercially available creams range between 70mg/d to 400mg/d per day, therefore results of this study may represent an “underdose” of transdermal Mg^2+^.

## Conclusion

No previous studies have looked at effects of transdermal Mg^2+^ in human subjects. In this two-week pilot study, transdermal delivery of 56 mg Mg/day (a low dose compared with commercial transdermal Mg^2+^ products available) showed a larger percentage change in both serum and urinary markers from pre to post intervention compared with subjects using the placebo cream. In addition, the rise in mean serum Mg^2+^ seen in the Mg^2+^ group was clinically relevant although only statistically significant (p<0.05) when non-athletes were analysed separately.

Given the high variance in serum Mg^2+^ of these subjects, we suggest that future research focus on a larger number of human subjects given higher concentrations of Mg^2+^ cream application administered for longer durations to investigate whether transdermal application may show a significant contribution to improvement in magnesium status. It would also be of interest to look at the effect of transdermal Mg^2+^ supplementation on athletes as compared to a sedentary population group.

## Supporting information

S1 Text Ingredients list. Ingredients for magnesium and placebo creams **list**.
(DOCX)

S1 Fig CONSORT checklist. Checklist of information to include when doing a randomised trial.
(DOC)

S1 Table Dataset for repository. Subject data.
(XLSX)

S2 Text Protocol (DOCX)
