# Supplementary material for: Effect of transdermal magnesium cream on serum and urinary magnesium levels in humans: A pilot study
Source: PLoS One. 2017 Apr 12;12(4):e0174817. doi: 10.1371/journal.pone.0174817 (PMC5389641; doi:10.1371/journal.pone.0174817)
Supplement: S1 Text — Ingredients for magnesium and placebo creams list. (DOCX) [file pone.0174817.s001.docx]

**S1 Text. Ingredients:** Ingredients for magnesium and placebo creams

**Magnesium Cream**: Lot # T10224:  Aqua, magnesium chloride (10%), cetearyl olivate, sorbitan olivate, isopropyl palmitate, emulsifying wax, glycerine, butyrospermum parkii (shea butter), hydroxypropyl starch phosphate, iodopropynyl butylcarbamate, phenoxyethanol, caprylyl glycol.

**Placebo Cream:** Emulsifying ointment (contains emulsifying wax, liquid paraffin and white soft paraffin. Emulsifying wax itself contains cetostearyl alcohol and sodium lauryl sulfate)

Phenoxyethanol , purified water
