# Supplementary material for: Effect of transdermal magnesium cream on serum and urinary magnesium levels in humans: A pilot study
Source: PLoS One. 2017 Apr 12;12(4):e0174817. doi: 10.1371/journal.pone.0174817 (PMC5389641; doi:10.1371/journal.pone.0174817)
Supplement: S2 Text — (DOCX) [file pone.0174817.s004.docx]

S2 Text Protocol and ethics

**UNIVERSITY OF HERTFORDSHIRE**

**Form EC1: Application for ETHICAL approval of a study involving human participants**
(See Guidance Notes)

**OFFICE USE ONLY**

**Protocol Number:**

**Initial classification:**

**Relevant ECDA:**

Science & Technology

Health & Human Sciences

Social Sciences, Arts & Humanities

x

| **Office Use only** | **Date Received by Clerk:** | | |  |
| --- | --- | --- | --- | --- |
| **Expedited Review** | | | | |
| Approved by Reviewer 1 *(sign & date)* | | | Approved by Reviewer 2 *(sign & date)* | |
| **Further Action:** *(tick appropriate box and provide details)* | | | | |
| Request Further Information | |  | Details: | |
| Refer for Substantive Review | |  |  |  |
| Refer for Full review | |  |  |  |
| Reject | |  |  |  |
| **Substantive Review** | | | | |
| Approved by Reviewer 1 *(sign & date)* | | | Approved by Reviewer 2 *(sign & date)* | |
| Approved by Reviewer 3 *(sign & date)* | | | Approved by Reviewer 4 *(sign & date)* | |
| **Further Action:** *(tick appropriate box and provide details)* | | | | |
| Request Further Information | |  | Details: | |
| Refer for Full review | |  |  |  |
| Reject | |  |  |  |
| **Full Review** | | | | |
| Request Further Information | |  | Details: | |
| Reject | |  |  |  |
| **CONFIRMATION OF APPROVAL**  **[To be completed by the Chairman or Vice-Chairman of the relevant ECDA, or by the Chairman of the University Ethics Committee – (**see GN 2.1.4)**]**  I confirm that this application has been approved by or on behalf of the committee named below.  Name/Sign…………………………………………………………Date……………………..  Name of committee ……………………... | | | | |

**DECLARATIONS**

| **DECLARATION BY APPLICANT** (See GN 2.1.3)   1. I undertake, to the best of my ability, to abide by accepted ethical principles in carrying out the study.   **(ii)** I undertake to explain the nature of the study and all possible risks to potential participants, to the extent required to comply with both the letter and the spirit of my replies to the foregoing questions (including information contained in Appendices 1 & 2).  **(iii)** Data relating to participants will be handled with great care. No data relating to named or identifiable participants will be passed on to others without the written consent of the participants concerned, unless they have already consented to such sharing of data when they agreed to take part in the study.  **(iv)** All participants will be informed **(a)** that they are not obliged to take part in the study, and **(b)** that they may withdraw at any time without disadvantage or having to give a reason.  Where the participant is a minor or is otherwise unable, for any reason, to give full consent on their own, references here to participants being given an explanation or information, or being asked to give their consent, are to be understood as referring to the person giving consent on their behalf. (See Q 19 above; also GN Pt. 3, and especially 3.6 & 3.7)  Enter your name here……Lindsy Kass…………………………..Date…18/03/14…………….  **If you are a member of staff, please obtain the signature of your line manager to indicate their agreement to this application:**  …………………………………………………… ………………………………………………………  (Signature) (Name in BLOCK CAPITALS and position within the School)  **DECLARATION BY SUPERVISOR** (see GN 2.1.3)  I confirm that the proposed study has been appropriately vetted within the School in respect of its aims and methods as a piece of research; that I have discussed this application for Ethics Committee approval with the applicant and approve its submission; and that I accept responsibility for guiding the applicant so as to ensure compliance with the terms of the protocol and with any applicable ethical code(s).  Enter your name here…………………N/A……………………………………...Date……………… |
| --- |

**Instructions for Applicants**

**Applicants are advised to read the Guidance Notes before completing this form.** Use of this form is mandatory [see UPR RE01, SS 7.1 to 7.3]. Your School may choose to add additional School specific questions in a separate appendix to the Form EC1. Please clarify with your Supervisor whether your School has chosen to append any extra questions.

Approval must be sought **and granted** before any investigation involving human participants begins [UPR RE01, S 4.4(iii)].

Abbreviations

GN=Guidance Notes

UPR=University Policies & Regulations

Q=Question

S=Section

SS=Sections

Pt =Part

**PLEASE NOTE:** Where alternative answers are offered, put a cross in the appropriate box.

X

For example: YES

Where a “write in” answer is requested, begin in the space provided below the question and continue as necessary**. All questions must be answered. Please answer in BLACK.**

1. **THE STUDY**

| **Q1.** Please give the title (or provisional title) of the proposed study. (NB – you will be asked for further details later)  The effects of transdermal magnesium cream on serum and urinary magnesium levels |
| --- |

1. **THE APPLICANT**

| **Q2.** Please answer **either** **Q2.1** **or** **Q2.2** by providing the information requested. **Q2.1** should be answered by individual applicants, both staff and students, who require protocol approval for work which they themselves intend to carry out. **Q2.2** should be answered by academic staff requiring approval for standard protocols governing classroom practical work (or equivalent work) to be carried out by a specified group of students. (See GN 2.2.1 & 2.2.19)  **Q2.1**. Name of applicant/(principal) investigator  Status:   1. undergraduate      1. postgraduate (taught/research)   x   1. academic staff   **(d)** other - please give details here      School/Department: Life and Medical Sciences    Programme of study or award (e.g. BA/MSc/PhD/Staff research): Staff  E-mail address: l.s.kass@herts.ac.uk    Name of supervisor: Lindsy Kass  Supervisor’s contact details (email, extension number): L.Kass@herts.ac.uk  **Q2.2**. **Class Protocol Applications Only.**  Name of applicant/(principal) investigator (member of staff)    School/Department  Programme of study or award (e.g. BSc/MA)  Module Title    Year/group to be governed by the protocol  Number of students conducting the study    Programme Tutor (if different from the applicant)  E-mail address  **Please note: Risk Assessment Form EC5 is mandatory for all Class Protocol Applications and must accompany this application.** |
| --- |

1. **DETAILS OF THE PROPOSED STUDY**

| **Q3. (a)** Is it likely that your application will require NHS approval? (See GN 2.2.2)  x  YES NO  (If YES, please answer **(b)** & **(c)**) (If NO, please continue on to **Q4**)    **(b)** Please confirm whether your research involves any of the following:    NHS Patients  Clinical trial of an investigational product  Clinical trial of a medical device  Exposure to any ionising radiation  Adults who lack the capacity to consent  Human Tissue  **(c)** Please confirm whether this study is considered to be a Clinical Trial of Investigational Medical Products (CTIMP) or Clinical Trial of Investigational Medical Devices. (See GN 2.2.2)  x  YES NO  If YES, please indicate if the study involves any of the following categories:  Children under 5  Pregnant women  A group of more than 5,000 people  Study would be undertaken overseas  **If your study is likely to require NHS approval or is a Clinical Trial of Investigational Medical Products or Devices, DO NOT complete this form any further and submit it to your relevant ECDA at this stage. All NHS applications must be made on an IRAS form. If your study is a Clinical Trial of Investigational Medical Products or Devices involving one of the above specified categories, you will be contacted by the relevant ECDA with information on the next steps. Please note, you will be issues with a UH Protocol Number but this will not be valid until you have sent your relevant ECDA a copy of your NHS approval.**  **Q4.** Please give a short synopsis of your proposed study; stating its aims and highlighting, if appropriate, where these aims relate to the use of human participants. (See GN 2.2.3)  Please enter details here.  The proposed study is an investigation to study the effects of magnesium cream on serum and urinary magnesium levels. The aim is to compare the results after the application of the topical cream to baseline measurements to see the absorption of the cream in order to identify if the cream could be used in future research in place of oral magnesium supplementation currently used.  **Q5.** Please give a brief explanation of the design of the study and the methods and procedures used, highlighting in particular where these involve the use of human participants. You should clearly state the nature of the involvement the human participants will have in your proposed study and the extent of their commitment. Thus you must complete and attach the Form EC6 (Participant Information Sheet) (see Appendix 2)**.** Be sure to provide sufficient detail for the Committee to be clear what is involved in the proposed study, particularly in relation to the human participants. (See GN 2.2.4)  This will be a randomised parallel design trial. Subjects will be recruited through the University of Hertfordshire and through word of mouth. Twenty participants will be given magnesium cream to be applied twice daily and 20 participants to be given a placebo (over the counter aqueous cream) to be applied twice daily. Two x 5ml teaspoons of cream to be applied daily for no more than 2 weeks with bloods and urine to collected at baseline and again after 2 weeks of application and tested for magnesium. The total application of magnesium cream will give a maximum of 56 milligrams of magnesium in 6 grams of cream or no magnesium in 6 grams of aqueous cream, twice The cream can be rubbed into any area of the body that does not have broken skin or an orifice. Food diaries and health questionnaires will be recorded over the period of the trial. Serum magnesium and 24h urinary magnesium excretion will be analysed.  **Q6.** Please give the starting date and finishing date. (For meaning of  “starting date” and “finishing date”, see GN 2.2.5)  Starting date: April 2014  Finishing date: April 2015  **Q7.** Where will the study take place? (If this is on UH Campus, who will permission be obtained from e.g. your Module Leader, Programme Tutor, Pro-Vice Chancellor (Student Experience) or the Dean of Students. If this is NOT on UH Campus, please attach a copy of the written permission, given by the proprietor, manager or other person with such authority over the premises, to use the premises for the purposes of carrying out this research (see Appendix 2)) (See GN 2.2.6)  Please enter details here.  The study will be conducted at the University of Hertfordshire. Permission to use the University of Hertfordshire’s human performance and human physiology laboratories (G111 and H260) has been obtained by the laboratory manager Neil Wilmore.  **Q8.** If the location is off campus, have you considered whether a risk assessment is necessary for the proposed location? (in respect of hazards/risks affecting both the participants and researchers) Please see **Form EC5** (see Appendix 2, which is an example of a risk assessment form.) Please use this example if a risk assessment is necessary, and you have not been provided with a subject specific risk assessment form by your School or Supervisor.  (See GN 2.2.7)  **Q9. (a)** Will anyone other than yourself and the participants be present with you when conducting this study? (See GN 2.2.8)  x  YES NO  If YES, please state the relationship between anyone else who is present other than the applicant and/or participants? (e.g. health professional, parent/guardian)  MSc by Research student Bradley Fleming will be present.  **(b)** Will the proposed study be conducted in confidence? If NOT, what steps will be taken to ensure confidentiality of the participants’ information. (See GN 2.2.8)  The information that is obtained during the study will follow ethical and legal practice and be handled in strict confidence. All paperwork will be placed within a folder which will be securely locked in a filing cabinet that is only accessible to the principal investigator or work supervisor. I will uphold individual’s anonymity, identity and take all reasonable steps to protect participants’ privacy, and to inform participations prior of any risk regarding identification in published material. All data will be made anonymous, therefore all figures and numbers will not be traceable. All paperwork will be shredded and all raw data will be deleted after the completion of study. |
| --- |

1. **HARMS, HAZARDS & RISKS**

| **Q10.** Will this study involve invasive procedures on the human participants? (See GN 2.2.9)  x  Yes No  (If YES, please fill out **Appendix 1 –** (If NO, answer **Q11, Q12, Q13**  **Increased Hazards and Risks.** **& Q14)**  Once this is complete, move on  to **Q15**)    **Q11, Q12, Q13 & Q14 - NON INVASIVE STUDIES ONLY**  Note: You are advised to read GN 2.2.10, 2.2.11, 2.2.12 & 2.2.13 carefully before you answer the following questions.  **Q11.** Are there potential hazards to participant(s) and/or investigator(s) from the proposed study? (See 2.2.10)  YES NO  If YES,   1. Indicate their nature here.      1. Indicate here what precautions will be taken to avoid or minimise any adverse effects.   **Q12.** Will or could the study cause discomfort or distress of a mental or emotional character to participants and/or investigator(s)? (See NG 2.2.11)  YES NO  If YES,   1. Indicate its nature here. 2. Indicate here what precautions will be taken to avoid or minimise such adverse effects.   **Q13.** Will or could medical or other aftercare and/or support be needed by participants and/or investigator(s) as a result of the study? (See GN 2.2.12)  YES NO  **Q14. (a)** If you have answered ‘YES’ to **Q11, Q12 & Q13**, please state here the previous experience (and/or any relevant training) of the supervisor (or academic member of staff applying for a standard protocol) of investigations involving the hazards, risks, discomfort or distress detailed in those answers. (See GN 2.2.13)  **(b)** Please describe in appropriate detail what you would do should the adverse effects or events which you believe could arise from your study, and which you have mentioned in your replies to the previous questions, occur.  (See UPR RE01, S 2.3 (ii) and GN 2.2.13) |
| --- |

1. **ABOUT YOUR PARTICIPANTS**

| **Q15.** Please give a brief description of the kind of people you hope/intend to have as participants, for instance, a sample of the general population, University students, people affected by a particular medical condition, children aged 5 to 7, employees of a particular firm, people who support a particular political party.  Participants will be male and female between the ages of 18 and 60  Participants will be recruited from the university and the surrounding area.  **Q16.** Please state here approximately how many participants you hope will participate in your study.  I wish to recruit 40 participants for the study which will be split in to 2 groups of 20.  **Q17.** By completing this form, you are indicating that you are reasonably sure that you will be successful in obtaining the number of participants which you hope/intend to recruit. Please outline here how you intend to recruit them. (See GN 2.2.14)  Participants will be recruited by posters, emails, social networking sites and verbally approaching individuals and groups. |
| --- |

1. **CONFIDENTIALITY AND CONSENT**

| [For guidance on issues relating to consent, see GN 2.2.15 & Pt. 3.]  **Q18.** Is it intended to seek informed consent from the participants?  x  YES NO  (See UPR RE01, S 2.3 & 2.4 and GN 3.1)  If NO, please explain why it is considered unnecessary or impossible or otherwise inappropriate to seek informed consent.  If YES, please attach a copy of the Consent Form to be used (See Form EC3 & EC4 for reference and GN 3.2), or describe here how consent is to be obtained and recorded. The information you give must be sufficient to enable the Committee to understand exactly what it is that prospective participants are being asked to agree to.  EC3  **Q19.** If the participant is a minor (under 18 years of age), or is otherwise unable for any reason to give full consent on their own, state here whose consent will be obtained and how? (See especially GN 3.6 & 3.7)  **Q20.** Are personal data of any sort (such as name, age, gender, occupation, contact details or images) to be obtained from or in respect of any participant? (See GN 2.2.16)  x  YES NO  If YES,   1. Give details here of personal data to be gathered, and indicate how it will be stored.   The personal data of each participant that will be gathered is; name, age, gender, date of birth, e-mail addresses and telephone number (contact purposes). Participants will be coded for anonymity. All data will be stored on a password protected computer and will not be accessible to anyone outside of the research team.  **(b)** State here what steps will be taken to prevent or regulate access to personal data beyond the immediate investigative team?  Personal data will be stored on a password protected pen drive and computer. No one other than the investigative team will be allowed access.  **(c)** Indicate here what assurances will be given to participants about the security of, and access to, personal data.  It will be made clear to the participants that their personal data will be kept confidential and stored away securely on a password protected pen drive and computer. Participants will be told that all personal information will be restricted to anyone who is not part of the research team. Also, they will be told that all personal information will be coded for anonymity purposes  **(d)** State here, as far as you are able to do so, how long personal data collected during the study will be retained, and what arrangements have been made for its secure storage.  Personal data will be retained until the examination process is finished. From the time the data is collected to when it is destroyed, all personal information will be kept secure on a password protected pen drive and computer.  **Q21.** Is it intended (or possible) that data might be used beyond the present study? (See GN 2.2.16)  x  YES NO  If YES, please give here an indication of the kind of further use that is intended (or which may be possible).  Publication in a scientific journal or presentation at a conference.  If NO, will the data be kept for a set period and then destroyed under secure conditions?  YES NO  If NO, please explain here why not.  **Q22.** If your study involves work with children and/or vulnerable adults you will require a satisfactory Enhanced Criminal Records Bureau Disclosure. (See GN 2.2.17) Please indicate as appropriate:  x   1. CRB Disclosure not required 2. CRB Disclosure required and obtained   If a satisfactory CRB Disclosure is required, a copy of this must be attached to Appendix 2 in order for reviewers to be able to consider your application. |
| --- |

1. **REWARDS**

| **Q23.** **(a)** Are you receiving any financial or other reward connected with this study? (See UPR RE01, 2.3)    x  YES NO  If YES, give details here.  **(b)** Are participants going to receive any financial or other reward connected with the study?  x  YES NO  If YES, give details here.  **(c)** Will anybody else (including any other members of the investigative team) receive any financial or other reward connected with this study?  x  YES NO  If YES, give details here. |
| --- |

1. **OTHER RELEVANT MATTERS**

| **Q24.** Enter here anything else you want to say in support of your application, or which you believe may assist the Committee in reaching its decision. |
| --- |

**APPENDIX 1 – INCREASED HAZARDS AND RISKS**

This section is to be completed if your answer to Q10 affirms the **USE OF INVASIVE PROCEDURES** in your study.

Note: You are advised to read GN 2.2.10, 2.2.11, 2.2.12, 2.2.13 & 2.2.18 carefully before you answer the following questions.

| **QA1.** Please give details of the procedures to be used (e.g. injection of a substance, insertion of a catheter, taking of a blood or saliva sample), and any harm, discomfort or distress that their use may cause to participants and/or investigator(s). (See GN 2.2.10)  Capillary and or venepuncture Blood Sample:   1. Indicate here what precautions will be taken to avoid or minimise any adverse effects.   All participants will have blood samples taken whilst sitting in a chair that is designed for blood taking. This will avoid the possibility of participants falling off of chairs in the event of losing consciousness whilst the sample is being taken. Health and Safety procedures will be followed when taking bloods, for example sterilisation of skin area and single usage needles  **QA2.** Will the study involve the administration of any substance(s)? (See GN 2.2.10)  x  YES NO  If YES,   1. Give details here of the substance(s), the dose or amount to be given, likely effects (including duration) and any potential hazards to participant(s) and/or investigator(s).   Magnesium cream containing 56mg of magnesium in 6 grams of cream per application for 2 weeks. There are no potential hazards to the participant or investigator beyond normal blood taking protocols that are carried out regularly.   1. Indicate here what precautions will be taken to avoid or minimise any adverse effects.   There should be no adverse effects however, participants will be sitting down when blood is collected and will be told to wait 5 minutes before leaving the laboratory.  **QA3.** Are there any potential hazards to participant(s) and/or investigator(s) arising from the use of the proposed invasive procedures? (See GN 2.2.10)  x  YES NO  If YES,   1. Indicate their nature here.   The potential hazards to the participant are being allergic to the hypoallergenic cream or the magnesium and the risk of infection originating from the site of the blood sample. The potential hazard to the investigator is infection from bloods.   1. Indicate here what precautions will be taken to avoid or minimise any adverse effects.   To reduce the risk of infection, the site of the blood sample will be thoroughly cleaned before the participant is lanced. Once the blood sample is complete, the participant will be given a plaster to cover the area in order to not allow any pathogens to enter the wound. The plaster will also provide a barrier between the participant and any surfaces or other people they may come into contact, therefore reducing the risk of others coming into contact with the participants blood. Gloves and a laboratory coat will be worn by the investigator as a further barrier between participant samples coming into contact with the investigator.  **QA4.** Will or could the study cause discomfort or distress of a mental or emotional character to participants and/or investigator(s)? (See GN 2.2.11)  x  YES NO  If YES,   1. Indicate its nature here 2. Indicate here what precautions will be taken to avoid or minimise such adverse effects.   **QA5.** Medical or other aftercare and/or support must be made available for participants and/or investigator(s) who require it where invasive procedures have been used in the study. Please detail what aftercare and/or support will be available and in what circumstances it is intended to be used. (See UPR RE01, S 2.3 (ii) and GN 2.2.12)  All participants will have the contact details of the investigator should they need further aftercare or support. The investigator will be available for them to contact should they have any questions or worries about the invasive procedures that have occurred. When blood samples have been taken all participants will be advised to remain in the chair until they feel well enough to stand. They will be supported by the investigator at this moment as there is the chance that a participant may feel unsteady on their feet if they do not like the sight of blood. In the event that throughout the testing the investigator feels there is a need for the participant to require further medical care, they will be advised to visit their GP and be referred on and not be allowed to take any further part in the study until they do so.  **QA6. (a)** Please state here previous experience (and/or any relevant training) of the supervisor (or academic member of staff applying for a standard protocol) of investigations involving hazards, risks, discomfort or distress as specified. (See GN 2.2.13)  The lead supervisor has previously completed several studies involving Mg supplementation, therefore has experience in giving participants supplementation.  **(b)** Please describe in appropriate detail what you would do should the adverse effects or events which you believe could arise from your study, and which you have mentioned in your replies to the previous questions, occur.  In the event of the participant getting an infection from the site of the blood sample, they will be advised to see their GP straight away in order to get the appropriate treatment. If they were to get cross contaminated with someone else’s blood on a recently injected sight, it would be thoroughly washed and participants would be told to go to the emergency unit of the local hospital to have tests completed to make sure there is no infection. If the investigator was contaminated by a participants blood or urine they will go to the accident and emergency unit of the local hospital.  **QA7.** In the event that the study reveals that a participant has a pre-existing medical condition (of which they may or may not be aware), and which could affect their present or future health or that of others, they should be informed of this in an appropriate manner and advised of follow-up action that they should take. (See GN 2.2.18) Advice should be sought as to whether information should be passed to their GP and a decision taken whether they should be allowed to continue to take part in the study. If a potential participant is not willing to agree to such action being taken in these circumstances, they should not be allowed to take part in the study. Please indicate here what arrangements have been made for complying with these requirements.  This will not be possible as information of this kind will not be collected.  **Please revert to Q15.** |
| --- |

**APPENDIX 2 – DOCUMENTS TO BE ATTACHED**

Please attach the following documents if you have affirmed possession of them in the relevant questions:

1. Permission from the location to be used to carry out this study (Q7)

x

(This includes permission to use a location on UH Campus and any

location off of UH Campus which requires permission to use.)

1. Risk assessment for off campus location (See Form EC5) (Q8)

x

1. Copy of Consent Form (See Form EC3 & Form EC4) (Q18)

x

1. Copy of Form EC6 (Participant Information Sheet) (Q5)
2. CRB Disclosure (Q22)
3. A copy of the proposed questionnaire and/or interview schedule (if appropriate for

this study). For unstructured methods, please provide details of the subject

areas that will be covered and any boundaries that have been agreed with

your Supervisor.
